# Design, Fabrication and Mass-spectrometric Studies of a Micro Ion Source for High-Field Asymmetric Waveform Ion Mobility Spectrometry

**DOI:** 10.3390/mi10050286

**Published:** 2019-04-27

**Authors:** Hua Li, Hongmei Yun, Xiaoxia Du, Chaoqun Guo, Ruosheng Zeng, Yongrong Jiang, Zhencheng Chen

**Affiliations:** School of Life and Environmental Sciences, GuiLin University of Electronic Technology, Guilin 541004, China; yun1472372895@126.com (H.Y.); 13997815375@163.com (X.D.); 18877382996@163.com (C.G.); zengrsh@guet.edu.cn (R.Z.)

**Keywords:** needle-to-cylinder, lithographie, galvanoformung and abformung (LIGA), ion source, high-field asymmetric ion mobility spectrometry (FAIMS)

## Abstract

A needle-to-cylinder electrode, adopted as an ion source for high-field asymmetric ion mobility spectrometry (FAIMS), is designed and fabricated by lithographie, galvanoformung and abformung (LIGA) technology. The needle, with a tip diameter of 20 μm and thickness of 20 μm, and a cylinder, with a diameter of 400 μm, were connected to the negative high voltage and ground, respectively. A negative corona and glow discharge were realized. For acetone with a density of 99.7 ppm, ethanol with a density of 300 ppm, and acetic ether with a density of 99.3 ppm, the sample gas was ionized by the needle-to-cylinder chip and the ions were detected by an LTQ XL™ (Thermo Scientific Corp.) mass spectrometer. The mass spectra show that the ions are mainly the protonated monomer, the proton bound dimer, and an ion-H_2_O molecule cluster. In tandem with a FAIMS system, the FAIMS spectra show that the resolving power increases with an increase in the RF voltage. The obtained experimental results showed that the micro needle-to-cylinder chip may serve as a miniature, low cost and non-radioactive ion source for FAIMS.

## 1. Introduction

In recent decades, chemical sample separation technology, based on the nonlinear characteristic of ion mobility at high and low electric field strengths, has developed rapidly. This technology is known as high-field asymmetric waveform ion mobility spectrometry (FAIMS) [1,2]. At present, there are mainly two types of FAIMS: Cylindrical FAIMS and planar FAIMS [3,4]. Compared with ion mobility spectrometry (IMS) and mass spectrometry (MS), FAIMS is better able to separate the isomers by using the characteristic that ion mobility would be changing at high electric fields (>10 kV/cm). Furthermore, FAIMS has many favorable characteristics, such as miniaturization, high sensitivity, short test time, numerous test substances, low power, etc., making it a good choice of analytical chemistry instruments [5,6,7].

FAIMS is mainly composed of four parts: the ionization source, the drift tube, the detector and the asymmetric RF waveform voltage. The function of the ion source is ionizing the neutral chemical sample into charged ions. In general, the ion source is the key component of FAIMS. The requirements of the FAIMS as the ion source are the following: high ionization efficiency, low electromagnetic radiation, miniaturization, etc. To our knowledge, several ion sources have been used in the FAIMS. UV lamp is one of the most commonly used ionization sources for FAIMS because of its low ionization energy, and it is also difficult to generate ion debris. However, low ionization energy limits the diversity of materials that can be ionized by FAIMS [8,9]. Most of the radioactive ion sources use ^63^Ni as the ionization source of the FAIMS system. It is characterized by stable performance, small size, high ionization energy and convenient to use. However, it has radioactive pollution and the ionization products are complex. This is not conducive to separation by the FAIMS system [10,11,12]. Electrospray ion sources have been used widely in mass spectrometers and are generally suitable for substances with strong ionization polarity [13,14]. X-ray is a commercially available ion source, which is not convenient to be miniaturized and integrated for the FAIMS system [15].

Another interesting ion source used for the FAIMS is the corona discharge ion source. The corona discharge ion source has been used successfully in IMS. However, the application of corona discharge ion source for FAIMS has seldom appeared, especially for the planar FAIMS. Guevremont et al. adopted the corona discharge ionization method for the cylindrical FAIMS (ion trapping at atmospheric pressure (760 Torr) and room temperature) [16]. Liu et al. designed a line-cylinder ion source based on the corona and glow discharge ion source [17]. This ion source can work at ambient pressure and room temperature. The line-cylinder is fabricated using microelectromechanical systems (MEMS) technology, but the discharge experiment has not been reported in the literature. Hence, the line-cylinder ion source has not been used in FAIMS. Zhao et al. fabricated a needle-mesh corona discharge ion source for use in FAIMS [18]. A dimethyl methylphosphonate (DMMP) sample was ionized and separated by the Corona Discharge (CD)-FAIMS system. On the other hand, the plasma ion source was used in tandem with FAIMS [19,20]. 

An attractive feature of the corona discharge ion source is the simple structure and possibility to be fabricated by MEMS technology. However, the MEMS discharge ion source for FAIMS has not been reported until now. The challenge of the MEMS ion source based on corona discharge is that it is not easy to get a stable discharge stage in micro distance. In addition, the interface between the ion source and the FAIMS chip should be designed carefully to keep air tightness. A small gas leakage will lead to experimental failure. 

The planar FAIMS system was easily fabricated by MEMS technology. The micro-machined FAIMS system was fabricated from two glass plates separated by 0.5 mm-thick silicon strips [21]. Unfortunately, the ion source was not miniaturized accordingly. The UV-lamp ion source and ^63^Ni ion source are usually used in the planar FAIMS system, which is not beneficial for FAIMS miniaturization. It is thus urgent to design and fabricate a MEMS ion source for the planar FAIMS chip.

In this paper, we report on the integration of a MEMS ion source within planar FAIMS. By spectrum experiments of the mass spectrometer and FAIMS, it shows that the MEMS ion source can ionize the chemicals into electrical ions and get the right FAIMS spectrum. Compared to other ion sources, the micro ion source has many advantages, such as low costs, miniaturization, simple structure, ease of integration with FAIMS, etc. The paper provides a new way to realize ion source function by MEMS technology, which is valuable for the miniaturization and integration of the FAIMS system.

## 2. Material and Methods

**Figure 1:**
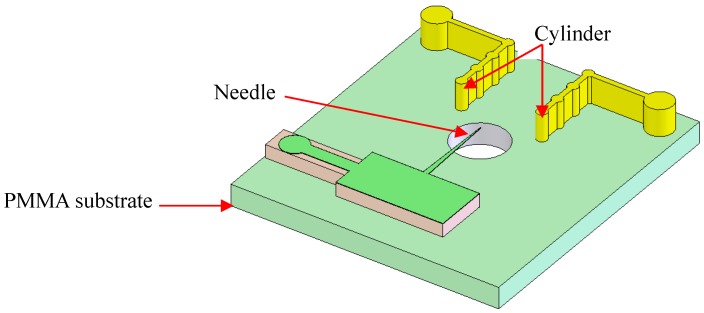
Schematic of the needle-to-cylinder micro ion source.

In a previous study, we showed that corona and glow discharge can be realized in an ambient atmosphere using the needle-to-cylinder electrode [22]. The COMSOL simulation results showed that the single central needle-to-double cylinder is the best structure of the four types of electrodes (single needle-to-single cylinder; single bottom needle-to-double cylinders; single central needle-to-double cylinders; and double needles-to-double cylinders) to achieve a stable and higher ion current under the same conditions. Therefore, the micro ion source design was designed and fabricated as a single needle-to-double cylinder configuration, shown in Figure 1. The needle and two-cylinder electrodes were formed on the PMMA substrate. There was a hole with a diameter of 0.75 mm for injecting the sample into the bottom substrate. When a high voltage was applied to the needle, the sample can be ionized by the air discharge process.

**Figure 2:**
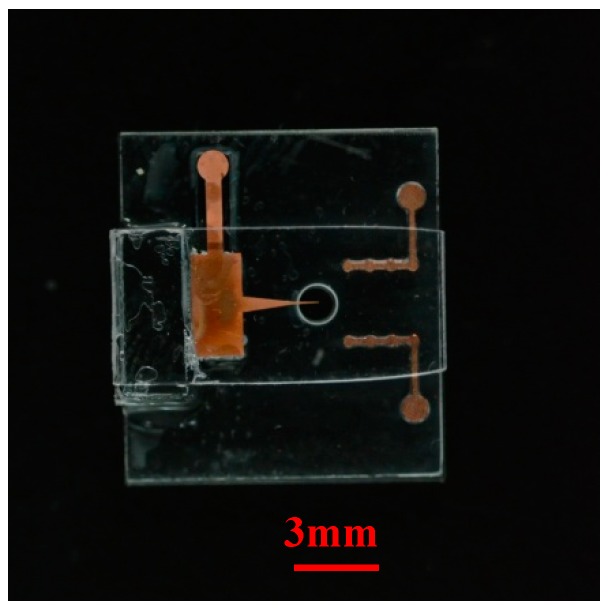
The micro ion source chip.

The fabrication process of the micro ion source has been described previously [23]. The needle and cylinder electrodes were fabricated by copper electroplating using LIGA (lithographie, galvanoformung and abformung) technology. A needle with a tip diameter of 20 μm and thickness of 20 μm was employed as the discharge cathode. Four cylinders with a diameter of 400 μm were connected to form the anode. The needle-to-cylinder electrode spacing was 2 mm. The dimensions of the chip were 11 mm × 10 mm × 2.3 mm, as shown in Figure 2. To cover the flow channel, the top substrate (PMMA) was attached on the cylinder electrodes. The top substrate and bottom substrate were cured and patterned using a patterning process. The conducting wires were welded in the solder joints to conduct the discharge experiments.

**Figure 3:**
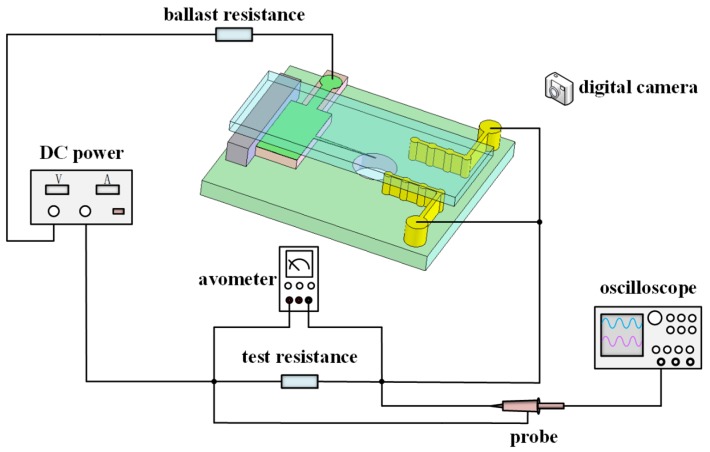
Experimental setup of the discharge circuit.

The micro ion source was operated as a negative DC discharge as this is more stable than a positive DC discharge. Figure 3 depicts the experimental setup of the micro ion source. A 0 to −5000 V, 500 W DC power supply was used for discharge power control. The discharge current was measured by the voltage of the test resistor of 1 kΩ. A 6 MΩ ballast resistor was connected to the needle to prevent the arc discharge. The actual AC discharge voltage was stored in a Tektronix oscilloscope and the voltage effective value was tested by a digital multimeter.

**Figure 4:**
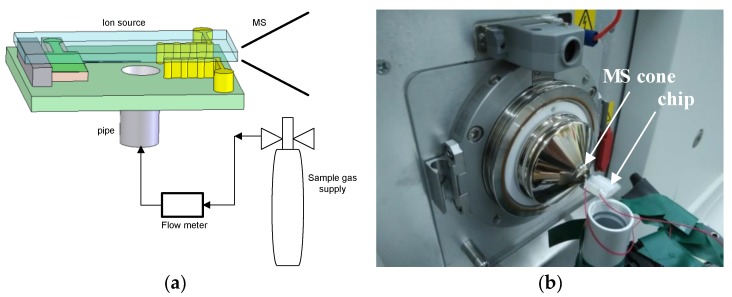
Schematic diagram of the chip-MS instrument. (**a**) Schematic of the experiment. (**b**) Experimental setup.

To obtain the ion characteristics of the sample, the chip was interfaced to the LTQ XL™ mass spectrometer (Thermo Scientific Corp., Waltham, MA, USA) The mass spectrometer worked in the positive ion mode and the range of charge to mass was chosen as 24 to 200. The work temperature was set as 30 °C and the voltage of the skimmer cone was 0 V. The mass spectrometer experimental setup is shown in Figure 4. To obtain the ion signal, the discharge voltage of the needle-to-cylinder electrode was −2500 V. The flow velocity of the sample gas was controlled by a flow meter (D08-1F provided by Beijing QixingHuachuang Electronics Co. Ltd., Beijing, China). With a pipe of 3 mm in diameter, the sample gas was injected into the ion source chip and ionized. An O-ring (Viton) was used to make a seal between the chip and the MS cone. Finally, the ions entered the MS cone and were detected according to their mass-to-charge ratios. The experimental setup was termed chip-MS.

## 3. Results and Discussion

### 3.1. The Discharge Characteristics

The discharge stability of the needle-to-cylinder electrode chip is very important as the ion source. The microchip works at ambient pressure and room temperature both with and without a N_2_ supply. First, the discharge starts when N_2_ is not supplied, which has been described in detail [23]. There were corona and glow discharges with the discharge voltage increasing from −3050 V to −3563 V.

**Figure 5:**
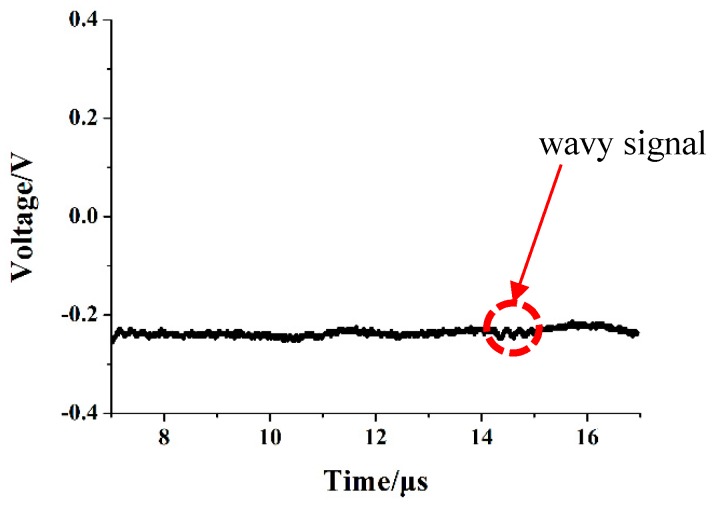
Discharge waveform with the N_2_ flow velocity of 1 L/min.

As the samples to be tested are volatile organic compounds, it is necessary to pass a carrier gas of a certain flow rate in the FAIMS system. With the carrier gas flow rate of 1 L/min and the discharge voltage of −2.5 kV, the discharge waveform is shown in Figure 5. After passing nitrogen, the microchip ion source enters the glow discharge stage directly. At this time, the pulse of the microchip ion source discharge waveform disappears, and the DC voltage signal appears. With the pin-to-column discharge voltage increasing, the DC signal also increases. 

In Figure 5, a wavy signal is shown. The wavy signal may come from discharge instability. Firstly, the high voltage DC power supply converts 220 V AC into DC high voltage. As there is a wavy signal in the 220 V AC, the wavy signal will also exist in the DC high voltage after a boost rectifier, which will result in the discharge instability. Secondly, the discharge plasma will damage the discharge electrode, especially the needle tip. With the discharge process ongoing, the damaged electrode will also affect the discharge stability. The observed wavy signal is similar to those observed in other studies [22,24].

**Figure 6:**
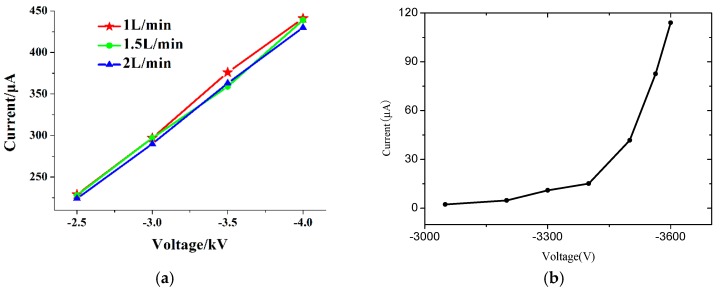
Discharge current with and without gas flow. (**a**) with different gas flow velocities; (**b**) without gas flow (see [23]).

The volt-ampere characteristic is shown in the Figure 6. Figure 6a shows the discharge characteristic of the ion source when the gas flow velocity is varied. Figure 6b concerns the discharge characteristic of the ion source without gas flow (see [23]). There are interesting findings when comparing our results with those found in [23]. The discharge current is almost the same under different N_2_ supply speeds, as shown in Figure 6a. This demonstrated that the gas jet does not have an obvious effect on the discharge current. In contrast to Figure 6b of [23], it shows different characteristics. In Figure 6b, there are corona and glow discharges, apparently, when there is no gas flow. In the stage of glow discharge, the current increases sharply [25]. But in Figure 6a, the experimental situation is different. The N_2_ is used as the carrier gas and there is gas flow in the discharge region. The volt-ampere characteristic is different from that in Figure 6b in [23]. Firstly, the corona discharge stage is difficult to be obtained with the gas flow. It converted directly into the glow discharge. A possible reason may be that, with the gas flow, the draft velocity of the negative ions increases. Accordingly, electron detachment from the negative ions also happens frequently. Additionally, the shielding effect of the negative ions has receded and finally disappeared. On the other hand, as the discharge gap between the needle and the cylinder is very short, the discharge emission can fill up the discharge gap easily. At this time, the discharge current becomes the DC current, which is completely derived from the electronic current [26,27]. Furthermore, the Trichel pulse (corona discharge) is hard to get. Secondly, the discharge current is almost the same under different N_2_ flow velocity. As the gas flow velocities are 1 L/min, 1.5 L/min and 2 L/min, the difference among these flow velocities are not obvious. Then the effect of the gas flow velocity on the electro detachment from the negative ions can be ignored. So, the discharge current is almost the same. For connecting with FAIMS, the discharge state of the ion source is almost the same with different gas flow velocities. 

### 3.2. MS Experiment

To show the ion species that was ionized by the ion source, mass spectrometry experiments were carried out using the experimental setup shown in Figure 4. The standard gas samples, such as acetone with a density of 99.7 ppm, ethyl acetate with a density of 300 ppm, and acetic ether with a density of 99.3 ppm, were all purchased from Beijing Hua Yuan Gas Chemical Industry Corporation. 

**Figure 7:**
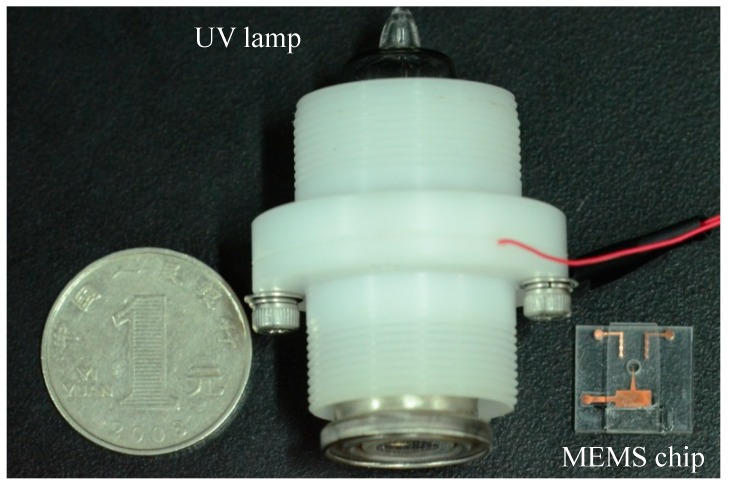
The UV lamp and the microelectromechanical systems (MEMS) chip.

Comparison of mass spectrometry experiments between needle-cylinder chip ion source and UV lamp ion source was carried out. The UV lamp was a 10.6 eV photo-discharge lamp (PKS106, Heraeus Company, Hanau, Germany), as shown in Figure 7. The whole dimension of the UV lamp was approximately φ20×50 mm, which was much larger than the chip (11 mm × 10 mm × 2.3 mm). 

On the other hand, the advantage of the MEMS technology is that it can fabricate in mass production. The MEMS chip was fabricated at the Institute of High Energy Physics, Chinese Academy of Science. A total of 50 MEMS chips were fabricated together. The unit price for a MEMS chip is about tens of dollars. The UV lamp (PKS106) used in the experiment was purchased from Heraeus Company, and the unit price is about hundreds of dollars. For a unit, a MEMS chip is much cheaper than the UV lamp.

**Figure 8:**
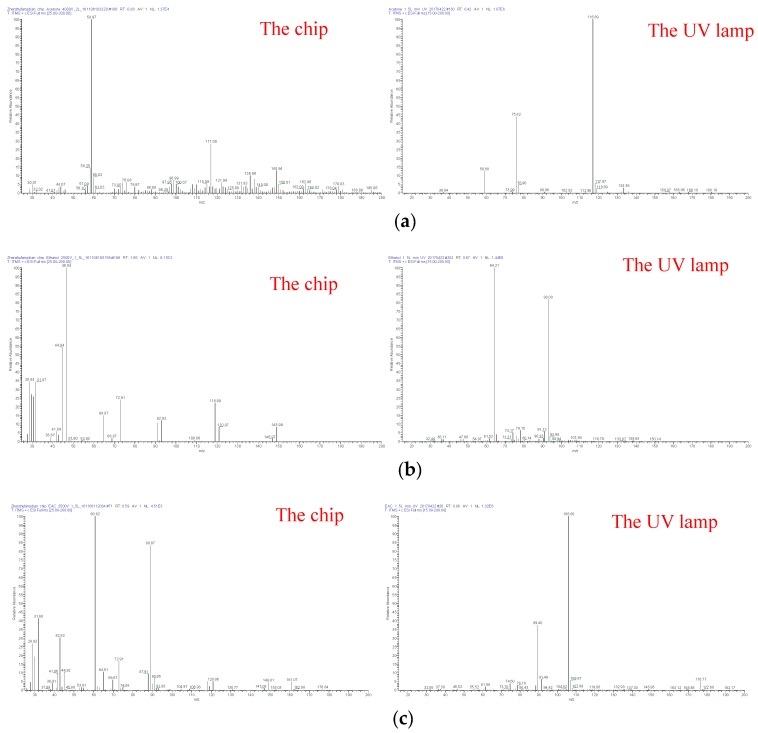
Comparison of mass spectra of (**a**) acetone (**b**) ethanol (**c**) acetic ether gas with the microelectromechanical systems (MEMS) chip and UV lamp ion source.

It is necessary to understand the type of reactant ions derived from the ion source [17,28,29]. Using the experimental setup in Figure 4, the UV-MS experiments were performed. In these experiments, the carrier gas flow velocity was set to 1.5 L/min. Compared with the spectra of the needle-cylinder chip-MS, the mass spectra of the UV lamp displayed in Figure 8 was simpler. The reason is that the UV ionization is a “soft” ionization method. However, the needle-cylinder chip ion source is based on air discharge, so the ionization energy is stronger than the ionization energy of the UV lamp ion source, thus ionizing more ion fragments.

A comparison of the results that were obtained by the chip and UV lamp ion source is summarized in Table 1.

Table 1 shows that the main ion species were almost the same for the chip and the UV lamp. The major ion species of acetone had m/z ratios of 59 and 117. For the chip, the highest peak was at 59 m/z, while the highest peak was at 117 m/z for the UV lamp. For ethanol, the peaks at m/z 93 and 64 were observed for the chip and UV lamp. However, the peak at m/z 47 was not observed for the UV lamp. The highest peak was at m/z 47 and m/z 64 for the chip and UV lamp, respectively. For acetic ether, the same peak was at m/z 89. However, the peaks at m/z 61 and 106 only existed for the chip and UV lamp, respectively. The highest peak was at m/z 61 and 106 for the chip and UV lamp. 

From the above analysis, it was obvious that the highest peak almost corresponded to the dimer or cluster for the UV lamp. However, the highest peak was related with the monomer for the chip. The possible reason is that the ionization efficiency is higher for the chip than that of the UV lamp. Hence, more gas sample exists after the UV lamp ion source than the chip. The gas sample is easily bound with the ions and dimers or clusters appear. The other reason is related to the delay time of the ion before entering the MS. As the volume of the UV lamp is larger than the chip, the connecter with the MS is also larger. Therefore, the longer the ions stay in the ionization chamber, the more dimers or clusters they will form. The monomer peak becomes less intense and the dimer or cluster peak becomes more intense for the UV lamp.

### 3.3. FAIMS Experiment

**Figure 9:**
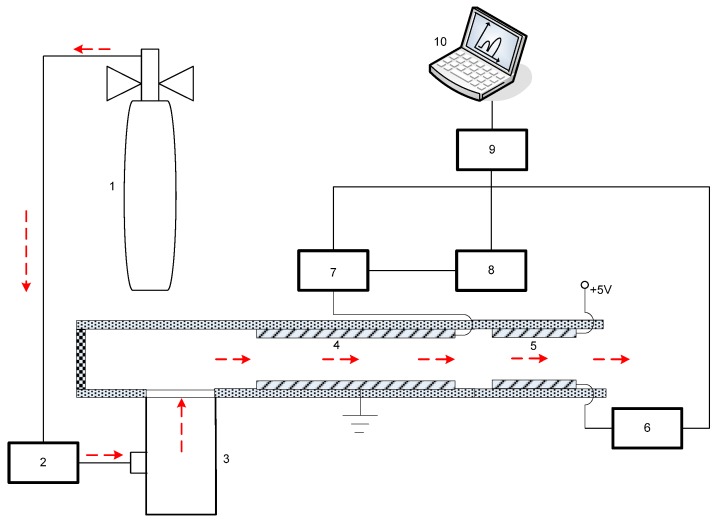
Schematic diagram of the high-field asymmetric ion mobility spectrometry (FAIMS) setup. 1. Sample gas; 2. Flow meter; 3. Microelectromechanical systems (MEMS) chip; 4. Drift region; 5. Detector; 6. Electrometer; 7. The high-filed rectangular asymmetric waveform; 8. The compensation voltage; 9. Controller; and 10. Computer.

In this work, the needle-cylinder MEMS chip was used as the ion source for the FAIMS. The schematic diagram of the home-made FAIMS experiment setup is shown in the Figure 9. The FAIMS consists of four parts: a needle-cylinder MEMS ion source, a drift region, a detector region, a high voltage radio frequency waveform and a low DC compensation voltage. The sample gases were 99.7 ppm acetone, 300 ppm ethanol and 99.3 ppm ethyl acetate. The flow velocity was maintained at 1.5 L/min by the flow meter (D08-1F, Beijing Sevenstar Electronics Co., Ltd., Beijing, China). A Teflon sheet was used as a 0.2 mm-gap spacer between the metal plates of the drift tube. The bottom metal plate was held at the ground electrical potential, while the high-field rectangular asymmetric waveform and the compensation voltage were applied to the upper metal plate. The upper electrode of the detector is connected to a +5 V deflection voltage, and the lower electrode is connected to an electrometer, and the charged ion signal is expressed in the form of current. The RF frequency, scope of compensation voltage, the scanning time and the start and end of the scanning were controlled by the controller. The FAIMS spectra data were stored and drawn with Labview software. 

As the MEMS chip is miniature, the gas tightness is very important for the FAIMS performance. The MEMS chip is connected to the FAIMS system by the glue to keep gas tightness. The FAIMS system is composed of two set of plates, which is fabricated by the standard printed circuit board (PCB) technology. A hole with 1.5 mm diameter in the one set of the plates is used as the ion and gas inlet from the MEMS chip. The two-way RF is used to generate the dispersion field. The differential-RF-driven operation mode can reduce power consumption, and increase signal-to-noise ratio [30]. 

The parameters of the FAIMS system are summarized in Table 2. 

According to the parameters in Table 2, the FAIMS experiment was successful. The RF voltage increased from 0 V to 400 V, while the compensation voltage was swept from +5 V to −10 V with the step size of 0.1 V. 

**Figure 10:**
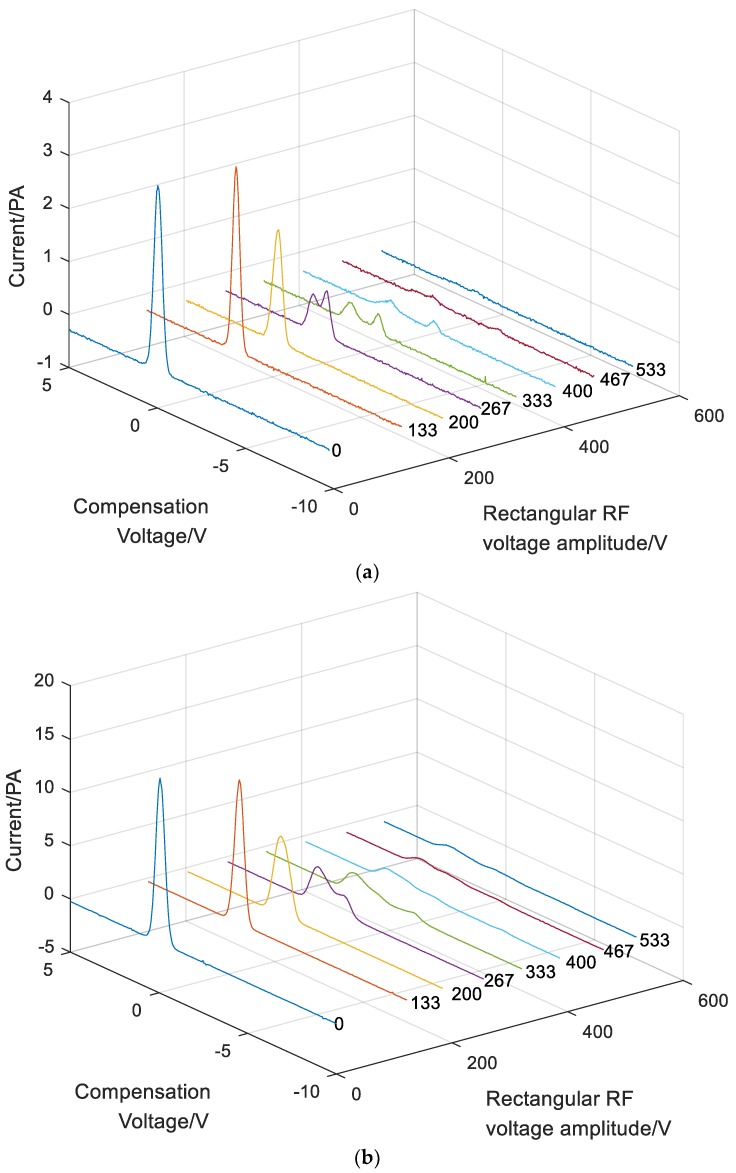

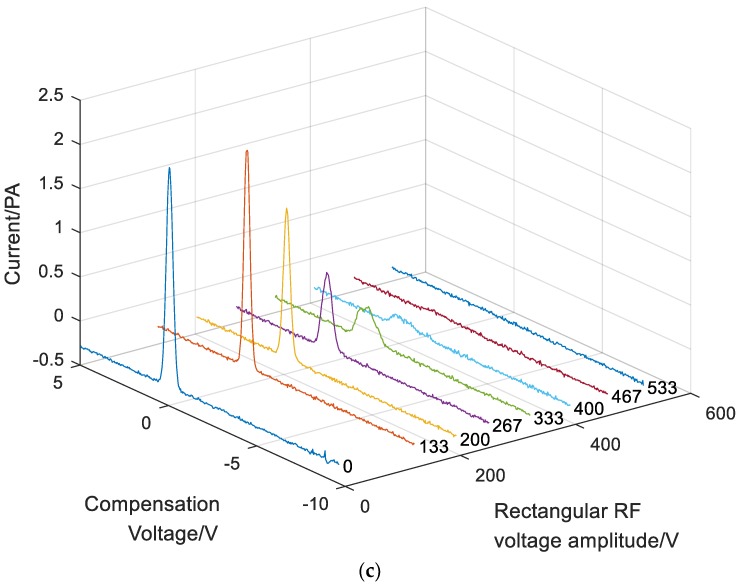
High-field asymmetric ion mobility spectrometry (FAIMS) spectra for acetone, ethanol and acetic ether with different applied radio frequency (RF) voltages. (**a**) Acetone; (**b**) Ethanol; (**c**) Acetic ether.

The FAIMS spectra for acetone, ethanol and acetic ether are presented in Figure 10. For each sample, the effects of the RF voltage on the spectra were analyzed. In this experiment, the flow velocity was constant at 1.5 L/min, while the RF voltage was increased. This shows that the intensity of the peak decreased with the increase of the RF voltage. This was expected as there was no compensation voltage needed when no electric field was applied across the drift tube. Hence, the maximum ion intensity will occur. With the increase in the applied RF voltage, the ions will scatter toward the electrodes of the drift tube under the RF electrical field. Hence, the ion intensity decreases, and the compensation voltage is needed to correct the trajectory of the ion species. There were two intensity peaks because the different ions are separated by the RF electrical field. The experiments showed that the needle-cylinder ion source could ionize the gas sample and obtain the corresponding FAIMS spectra.

## 4. Conclusions

A MEMS chip of a needle-cylinder electrode has been successfully fabricated. The ability to ionize the sample and integrate FAIMS is demonstrated by the MS ion species and FAIMS separation experiments. The experimental results of MS are more complex than those of UV lamps. However, the main ion species, such as protonated monomer and dimer, are almost identical. If the needle-cylinder chip ion source is applied to the FAIMS system, the function of ionization and detection of substances in the FAIMS system can be realized. Compared with the UV lamp, the MEMS needle-cylinder ion source has the characteristics of low cost, miniaturization, high sensitivity, ease of integration, etc. The attractive features of the MEMS chip are its micro volume and stable signal. The above features cannot be realized with a UV lamp. The other attractive features of the air discharge ion source are that the ionization intensity can be adjusted by varying the parameters of discharge voltage and the integration of ion source within the FAIMS system. This result is not found in the present literature. The experimental results will be shown in a following paper.

## Figures and Tables

**Table 1:** Ion species in mass spectra obtained by the needle-cylinder chip and UV lamp.

Gas Sample	Molecular Weight	Peaks				
		Ionization Method	Monomer	Dimer	Cluster	Fragments
acetone	58	The chip	59[MH^+^] *	117[M_2_H^+^]	60,73,136,149	30
		UV lamp	59[MH^+^]	117[M_2_H^+^] *	76[M+H_2_O]	
ethanol	46	The chip	47[MH^+^] *	93[M_2_H^+^]	64[M+H_2_O],73,93,119,121,149	29,32,45,
		UV lamp		93[M_2_H^+^]	64[M+H_2_O] *	
acetic ether	88	The chip	88,89[MH^+^], 91,61[M+H^+^-CO] *			29,32,43,73,
		UV lamp	89[MH^+^]		106[M+H^+^+N_3_H] *	

* The highest peak.

**Table 2:** Parameters of the high-field asymmetric ion mobility spectrometry (FAIMS) system.

Features	Dimensions	Features	Dimensions
Curvature radius of needle	10 μm	Thickness of needle	20 μm
The drift tube	10 mm × 5 mm	The whole dimension of the chip	11 mm × 10 mm × 2.3 mm
The radio frequency (RF)	1 MHz	The RF max voltage	0~400 V
The gap distance of the drift tube	0.2 mm	The voltage of the detector	5 V
The detector	5 mm × 5 mm	The scope of compensation voltage	−10 V~ +5 V
Ion source	The chip	The carrier gas flow	1.5 L/mm
